# Enhanced Molecularly Imprinted Fluorescent Test Strip for Rapid and Visual Detection of Norfloxacin via a Smartphone

**DOI:** 10.3390/molecules29030661

**Published:** 2024-01-31

**Authors:** Bo Hu, Wenshi Zhao, Li Chen, Yang Liu, Zhongfei Ma, Yongsheng Yan, Minjia Meng

**Affiliations:** 1 School of Environment and Safety Engineering, Jiangsu University, Zhenjiang 212013, China; 2111809001@stmail.ujs.edu.cn (B.H.); mzf@ujs.edu.cn (Z.M.); 2 Key Laboratory of Functional Materials Physics and Chemistry of the Ministry of Education, Jilin Normal University, Changchun 130103, China; zws6979@163.com (W.Z.); liuyang@jlnu.edu.cn (Y.L.); 3 School of Chemistry and Chemical Engineering, Jiangsu University, Zhenjiang 212013, China; lchen@ujs.edu.cn (L.C.); yys@mail.ujs.edu.cn (Y.Y.)

**Keywords:** norfloxacin, fluorescent test strip, molecularly imprinted polymers, on-site visual detection, smartphone

## Abstract

Paper-based test strips with on-site visual detection have become a hot spot in the field of target detection. Yet, low specific surface area and uneven deposition limit the further application of test strips. Herein, a novel “turn-on” ratio of molecularly imprinted membranes (Eu@CDs-MIMs) was successfully prepared based on a Eu complex-doped polyvinylidene fluoride membrane for the selective, rapid and on-site visual detection of norfloxacin (NOR). The formation of surface-imprinted polymer-containing carbon dots (CDs) improves the roughness and hydrophilicity of Eu@CDs-MIMs. Fluorescence lifetimes and UV absorption spectra verified that the fluorescence enhancement of CDs is based on the synergistic effect of charge transfer and hydrogen bonding between CDs and NOR. The fluorescent test strip showed a linear fluorescent response within the concentration range of 5–50 nM with a limit of detection of 1.35 nM and a short response time of 1 min. In comparison with filter paper-based test strips, Eu@CDs-MIMs exhibit a brighter and more uniform fluorescent color change from red to blue that is visible to the naked eye. Additionally, the applied ratio fluorescent test strip was combined with a smartphone to translate RGB values into concentrations for the visual and quantitative detection of NOR and verified the detection results using high-performance liquid chromatography. The portable fluorescent test strip provides a reliable approach for the rapid, visual, and on-site detection of NOR and quinolones.

## 1. Introduction

Quinolones, especially fluoroquinolones, are widely used to treat various diseases caused by bacterial infections due to their strong antibacterial properties [1,2]. Among them, norfloxacin (NOR), as the first clinically used fluoroquinolone, has been applied to treat various diseases such as urinary and reproductive system infections [3], respiratory infections [4], and intestinal infections [5]. However, the long-term use of NOR is bound to result in its residue in the environment, which in turn poses potential risks to the environment and ecosystems and thus causes reproductive toxicity to aquatic lifeforms [6], antibiotic resistance [7] and joint toxicity [8]. In consideration of the widespread distribution and seasonal variations of NOR in aquatic environments [9,10], it is necessary to detect NOR more frequently. At present, various laboratory instrument-based methods have been used for the determination of NOR, including electrochemical detection [11], enzyme-linked immunosorbent assay (ELISA) [12], the surface-enhanced Raman scattering (SERS) method [13] and high-performance liquid chromatography (HPLC) [14]. However, the high specificity and sensitivity of the above methods often require professional operation, expensive instruments, and intricate pre-treatment processes [15,16,17], all of which are not conducive to long-term monitoring of NOR residues. Therefore, it is necessary to establish an effective method for portable, highly sensitive, and rapid determination of NOR in water environments. 

At present, the ratio fluorescence technology with visualization function has been developed to achieve the on-site detection of NOR [18], which enables a high-sensitivity measurement of targets by measuring the fluorescence intensity ratio of two fluorescent materials in the sensing system. Among many fluorescent materials, europium complexes are suitable for use as reference signals because of their broad excitation band, narrow emission band and long fluorescence lifetime [19]. Additionally, to achieve the selective detection of NOR in complex environments, it is essential to introduce materials with specific recognition functions into fluorescence sensors. The molecular imprinting technique (MIT) is an effective method to efficiently recognize imprinted molecules by constructing biomimetic synthetic receptors. The fluorescent imprinting sensors can be employed to provide visual detection without the usage of any instruments by introducing molecular imprinting polymers (MIPs) with specific recognition capabilities [20,21]. Traditional fluorescent imprinting sensors typically require dispersion in aqueous or organic solutions to complete the detection process [22]. Compared with the traditional nano-powder materials, convenience and practicality are the hallmarks of pH-inspired paper-based fluorescent test strips, which can achieve more intuitive on-site detection [23].

Nowadays, the impregnation method is mostly used to manufacture paper-based fluorescent test strips, which are obtained by simply dispersing the fluorescent sensor dispersion on the filter paper [24]. Although fluorescent sensors are more uniformly deposited on filter paper substrates by vacuum filtration [25] and ink-jet printing [26], the limitations of physical deposition methods still lead to a low reliability and poor accuracy of fluorescent test strips. Moreover, the low specific surface area of the filter paper substrate may hinder internal mass transfer, thereby impairing detection performance [27]. Therefore, the key to addressing the limitations of paper-based fluorescence test strips is to choose suitable substrate materials [28,29]. Polymer membranes have been used to produce fluorescent test strips to enhance visualization and detection performance. Polymer membranes-based fluorescent test strips are typically produced from pre-prepared imprinted polymers by vacuum filtration [30] or blending [31]. Interestingly, Zhang et al. prepared imprinting materials into a fiber membrane using the electrostatic spinning method [32]. However, MIPs embedded in the matrix membrane tend to interfere with binding of the target analyte and reduce the sensitivity and visualization of detection [33,34]. Therefore, mixing reference signals to prepare a substrate membrane and using fluorescent monomers as response signals to construct fluorescent test strips can improve detection sensitivity and reduce background interference.

**Figure 1:**
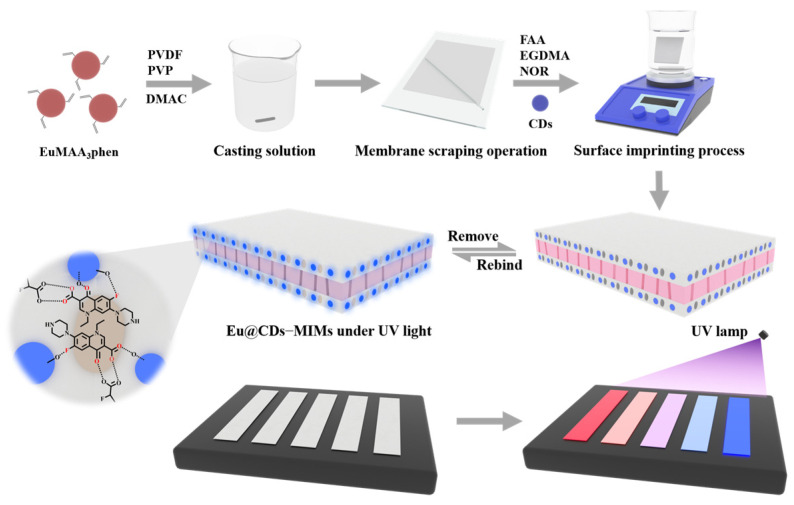
The schematic diagram of Eu@CDs-MIMs synthesis and visual detection.

In this work, a novel “turn-on” fluorescent test strip based on a PVDF membrane doped with europium complexes was successfully prepared to achieve the rapid visual detection of NOR in water. A highly luminescent europium complex Eu(MAA)_3_phen was dissolved in a casting solution and uniformly dispersed in the matrix membrane as a fluorescence reference signal by the phase conversion method. The molecularly imprinted membrane (MIM) and non-imprinted membrane (NIM) as fluorescent test strips (Eu@CDs-MIMs/NIMs) were prepared by the one-step method on the surface of Eu(MAA)_3_phen-doped PVDF membranes, as shown in Figure 1. Non-toxic carbon dots (CDs) and 2-fluoroacrylic acid (FAA) were used as the fluorescent response signal and functional monomer in the imprinting process, respectively. The fluorescence-enhanced NOR detection system was constructed based on the strong hydrogen bond interaction between CDs and NOR [35]. The fluorescence analysis results demonstrated that the imprinting process on the surface of the substrate membrane improved the hydrophilicity of the Eu@CDs-MIMs and shortened the response time to NOR. Under the optimal conditions, red-to-blue visualization was displayed in the range of 5–50 nM with a detection limit of 1.35 nM. Furthermore, a smartphone was used to enhance color-based quantitative analysis and compare it to HPLC. The results showed that Eu@CDs-MIMs were capable of providing rapid visual detection of NOR in situ.

## 2. Results and Discussion

### 2.1. Characterization of Eu@CDs-MIMs

**Figure 2:**
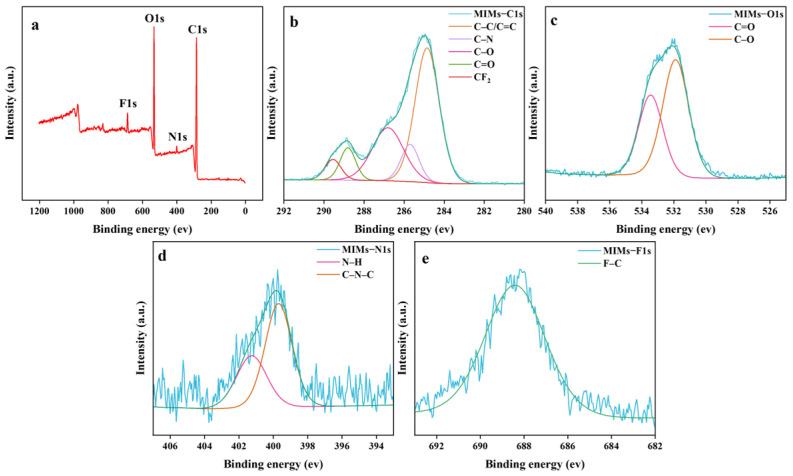
XPS survey spectra of Eu@CDs-MIMs (**a**) and high-resolution XPS spectra for C 1s (**b**), O 1s (**c**), N 1s (**d**) and F 1s (**e**).

The surface chemical composition of Eu@CDs MIMs was further evaluated by XPS spectra analysis. The survey spectrum reveals the presence of C, O, N and F elements in Eu@CDs-MIMs, as shown in Figure 2a. The C 1s peak positions located at 284.8, 285.7, 286.81, 288.8 and 289.5 eV are ascribed to C–C/C=C, C–N, C–O, C=O and -CF_2_, respectively (Figure 2b). As shown in Figure 2c, the high-resolution spectra of O 1s at 531.8 and 533.4 eV stem from C–O and C=O. The high-resolution spectra of N 1s (Figure 2d) consist of 399.7 and 401.2 eV, which are attributed to the typical N–C and N–H groups of CDs in the imprinted layer. The high-resolution spectra of F 1s (Figure 2e) indicate the presence of an F–C group (688.4 eV). In summary, the XPS analysis results are consistent with the FT-IR results.

The infrared spectra of PVDF, Eu@PVDF, and Eu@CDs-MIMs are shown in Figure S1. All samples show the characteristic absorption peaks of -CF_2_ and -CH_2_ at 1176 and 1403 cm^−1^ from PVDF, which agree with the results in references [36]. The feature peak at 1637 cm^−1^ is attributed to the C=C stretching from MAA, which confirms that Eu(MAA)_3_phen is successfully doped into the PVDF membrane and can be used for subsequent surface-imprinting processes. The feature peak at 1736 cm^−1^ belongs to the C=O stretching vibration of carboxyl from EGDMA, indicating that the imprinted layer with EGDMA as the crosslinking agent is successfully polymerized on the PDVF surface. All the results confirm that Eu@CDs-MIMs is successfully prepared. In summary, FT-IR analysis results are consistent with XPS results.

**Figure 3:**
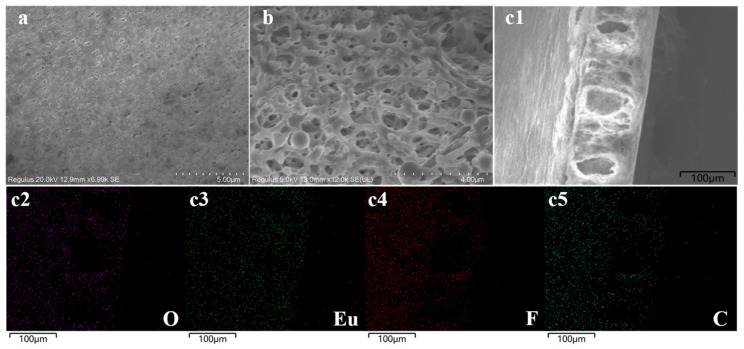
SEM images of Eu@PVDF (**a**), Eu@CDs-MIMs (**b**), and EDS mapping images of Eu@PVDF (**c1**–**c5**).

Figure 3 shows the SEM images of Eu@PVDF and Eu@CDs-MIMs. Figure 3a,b show that the Eu@CDs-MIMs have different surface morphology compared with the Eu@PVDF, and a noticeable polymer layer appears. This indicates that the imprinted layer on the surface of Eu@PVDF has been successfully polymerized. Energy-dispersive spectrometer (EDS) mapping images of Eu@PVDF (Figure 3(c1–c5)) illustrate the successful doping and homogeneous dispersion of Eu(MAA)_3_phen in PVDF membranes, which facilitates the uniform distribution of the imprint layer on the surface of Eu@PVDF. Color differences due to uneven loading are improved so that the visualization of the Eu@CDs-MIMs will be closer to the detection in solution.

**Figure 4:**
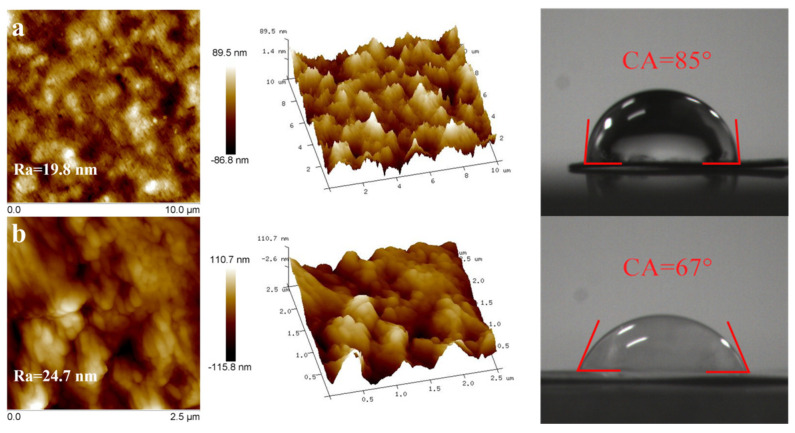
Two-dimensional and three-dimensional surface AFM and water contact angle images of Eu@PVDF (**a**) and Eu@CDs-MIMs (**b**).

The surface AFM and water contact angle images of Eu@PVDF and Eu@CDs-MIMs are shown in Figure 4. The surface structure of Eu@PVDF appears relatively smooth and flat with circular voids (Figure 4a). After the surface imprinting process is completed, the polymer layers modify the surface microstructures of Eu@PVDF and improve the roughness of Eu@CDs-MIMs (Figure 4b). In addition, the change in water contact angle from 85° to 67° confirms that the imprinted layer enhances the hydrophilicity of the MIMs, which improves the detection and visualization effect of the test strips.

### 2.2. Optimization of the Molar Ratio of NOR to FAA

**Figure 5:**
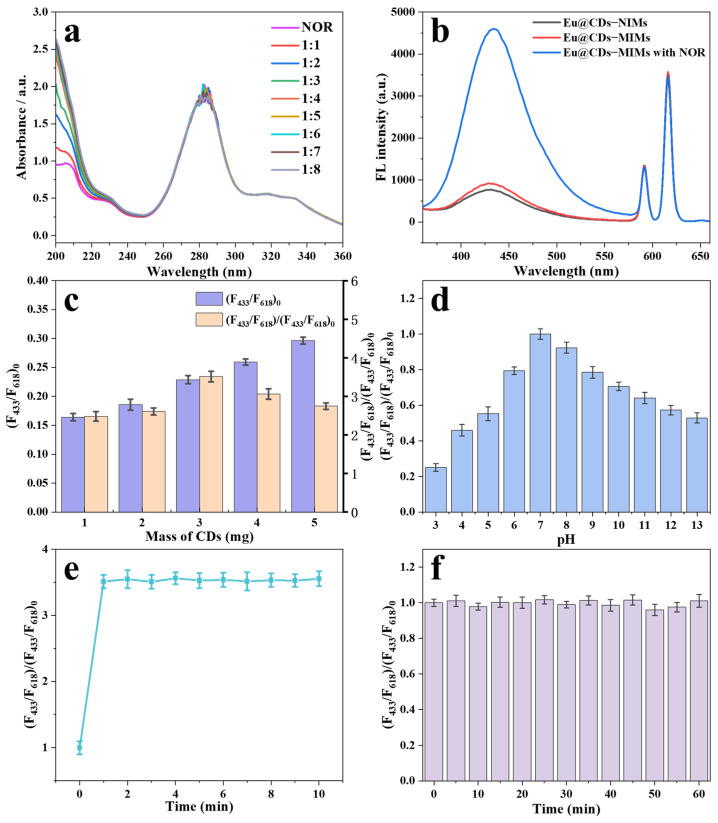
UV absorption spectrum of NOR and NOR-FAA mixtures (**a**). Fluorescence spectra of Eu@CDs-NIMs (black line), Eu@CDs-MIMs (red line) and Eu@CDs-MIMs with NOR (blue line) (**b**). (F_433_/F_618_)_0_ and (F_433_/F_618_)/(F_433_/F_618_)_0_ with different doping amounts of CDs (**c**). Effect of pH on fluorescence intensity of Eu@CDs-MIMs (**d**). The incubation time of Eu@CDs-MIMs to NOR (**e**). Fluorescence intensity changes of Eu@CDs-MIMs within 60 min (**f**).

The specific recognition ability of MIPs is mainly achieved through the interaction between template molecules and functional monomer bonds [37]. Therefore, a UV spectrophotometer was utilized to measure the absorbance of NOR and NOR-FAA mixtures in acetonitrile solution with different molar ratios to determine the optimal preparation conditions. As shown in Figure 5a, two prominent absorption peaks at 210 and 284 nm are observed in the UV spectrum, which are attributable to the π–π* transition of the carbonyl and benzene ring in NOR, respectively [38]. After the addition of FAA, there is no significant change in the absorption peaks at 284 nm, indicating that the benzene ring in NOR has no interaction with functional monomers. Instead, the absorption peak at 210 nm significantly increases with the increase in FAA and then remains relatively unchanged until the molar ratio reaches 1:4. As a result, the optimal molar ratio of NOR to FAA is determined to be 1:4.

### 2.3. Optimization of the Excitation Wavelength

The fluorescence spectra of Eu@CDs-NIMs (black line), Eu@CDs-MIMs (red line) and Eu@CDs-MIMs with NOR (blue line) are shown in Figure 5b. The sharp emission peak at 618 nm is due to the red emission of Eu(MAA)_3_phen that is doped into PVDF, whereas the broad peak centered at 433 nm is associated with the blue emission of CDS. After combination with NOR, the blue emission of Eu@CDs-MIMs is significantly enhanced, and the red emission as a reference signal remains unchanged. The smaller difference in fluorescence intensity between Eu@CDs-MIMs and Eu@CDs-NIMs is attributed to the hydrogen bonding interaction between NOR and CDs. This suggests that Eu@CDs-MIMs enable a ratio fluorescence detection for NOR through fluorescence enhancement. To determine the optimal detection wavelength for Eu@CDs-MIMs, the excitation dependence of Eu(MAA)_3_phen and CDs was evaluated and is shown in Figure S3. The optimal excitation wavelengths of Eu(MAA)_3_phen and CDs are located at 350 and 344 nm, respectively. In view of the extremely strong fluorescence enhancement of CDs during the detection process, the optimal excitation wavelength has been determined to be 344 nm, which is close to the commonly used 365 nm excitation wavelength of small commercial UV lamps.

### 2.4. Optimize Doping Amount of CDs

The performance of fluorescence detection and visualization effects can affected by the amount of CDs doping in the Eu@CDs-MIMs. Excess CDs will produce aggregation-induced quenching (ACQ) phenomena due to π–π interactions and resonance energy transfer causing fluorescence quenching [39]. However, a low doping amount of CDs cannot produce satisfactory fluorescence enhancement. In order to obtain the optimal doping amount of CDs, (F_433_/F_618_)_0_ and (F_433_/F_618_)/(F_433_/F_618_)_0_ of Eu@CDs-MIMs after binding NOR (50 nM) with different doping amounts of CDs (1, 2, 3, 4, 5 mg) are shown in Figure 5c. When increasing the doping amount of CDs, the (F_433_/F_618_)_0_ also increases gradually. In contrast, the (F_433_/F_618_)/(F_433_/F_618_)_0_ reaches the maximum when the doping amount of CDs is up to 3 mg, and then it shows a decreasing trend. Therefore, the optimal doping amount of CDs is determined to be 3 mg to achieve optimal detection sensitivity and visualization performance.

### 2.5. Optimization of the Detection Conditions

The detection environment, response time and stability are prerequisites and important influencing factors for performance testing. In order to confirm the applicability of Eu@CDs-MIMs as a test strip in different pH water environments, the fluorescence performance of Eu@CDs-MIMs was evaluated over a range of 3.0–13.0. The results in Figure 5d indicate that the relative fluorescence intensity is significantly reduced under acidic conditions, which is attributable to the fluorescence quenching of CDs under acidic conditions. Moreover, hydrogen bonding is reduced by hydrogen ions in solution in an acidic medium, which affects the hydrogen bonding between FAA and NOR. Additionally, the fluorescence enhancement of CDs based on hydrogen bonding is also weakened [40]. The Eu@CDs-MIMs exhibits good fluorescence stability under alkaline conditions. As a result, the optimal pH to detect NOR in water is pH 7. The changes in fluorescence intensity for different incubation times of Eu@CDs-MIMs to NOR (50 nM) were recorded and are shown in Figure 5e. The fluorescence intensity of Eu@CDs-MIMs rapidly increases to its highest value within 1 min and remains stable in the subsequent time. Consequently, the response time of Eu@CDs-MIMs is chosen to be 1 min. The fluorescence stability of Eu@CDs-MIMs was evaluated by continuous fluorescence detection 13 times and is shown in Figure 5f. The results suggest that Eu@CDs-MIMs has sufficient fluorescence stability to complete all visual test processes within 60 min.

### 2.6. Fluorescence Detection of Eu@CDs-MIMs and Eu@CDs-NIMs

**Figure 6:**
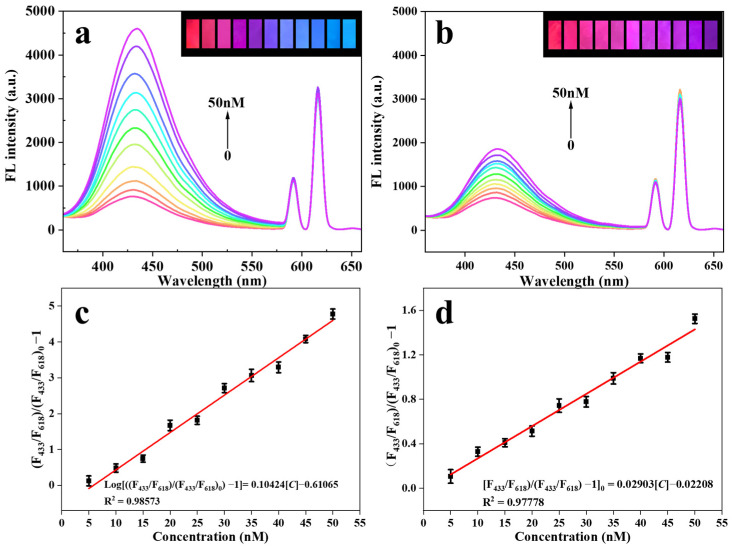
Fluorescence spectra of Eu@CDs-MIMs (**a**) and Eu@CDs-NIMs (**b**) in the concentration range of NOR from 0 to 50 nM. Linear relationship of Eu@CDs-MIMs (**c**) and Eu@CDs-NIMs (**d**).

The fluorescence detection performance of Eu@CDs-MIMs and Eu@CDs-NIMs was investigated under optimal conditions. Both Eu@CDs-MIMs and Eu@CDs-NIMs were fully immersed in the NOR solution to be tested in the concentration range of 0–50 nM for 1 min and then used for following detection. As shown in Figure 6a, the fluorescence intensities of Eu@CDs-MIMs at 433 nm are gradually enhanced with the increase in NOR concentration, while the reference signal at 618 nm remains unchanged. The fluorescence intensity of Eu@CDs-NIMs at 433 nm (Figure 6b) has the same enhancement trend, but the magnitude is much smaller than Eu@CDs-MIMs. This phenomenon can be ascribed to the specific recognition sites formed during the MIPs preparation process, and the selective binding of Eu@CDs-MIMs to NOR in solution is enhanced by the presence of specific recognition sites, which improves the fluorescence intensity at 433 nm through hydrogen bonding between NOR and CDs.

To further investigate the fluorescence enhancement of Eu@CDs-MIMs in the NOR concentration range, the linear relationship between the fluorescence intensity ratio of (F_433_/F_618_)/(F_433_/F_618_)_0_ and the concentration of NOR was determined using Stern–Volmer equations. As shown in Figure 6c,d, both Eu@CDs-MIMs and Eu@CDs-NIMs exhibit excellent linear relationships of (F_433_/F_618_)/(F_433_/F_618_)_0_ within the 0–50 nM range. The linear equation for Eu@CDs-MIMs is Log[(F_433_/F_618_)/(F_433_/F_618_)_0_ − 1] = 0.10424[C] − 0.61065 with an R^2^ of 0.98573. The imprinting factor (IF) (IF = kMIP/kNIP) [41] is calculated to be 3.27, and the limit of detection (LOD) is determined to be 1.35 nM by the equation LOD = *3σ*/*k* (*n* = 10) in which σ represents the slope of the linear equation, *σ* represents the relative standard deviation (RSD) of Eu@CDs-MIMs after detecting 10 times, and *n* is the number of detection times. Additionally, the limit of quantitation (LOQ) is found to be 4.5 nM (LOQ = 10*σ*/*k*). As a comparison, SiO_2_-based Eu/CDs-MIPs also have a good linear relationship, as shown in Figure S4. Because Eu(MAA)_3_phen embedded in MIPs tend to reduce the fluorescence efficiency and may interfere with target analyte binding, a higher LOD of 7.43 nM is achieved [34]. The results demonstrate the availability of Eu@CDs-MIMs for the detection of NOR within a concentration range of 5–50 nM.

### 2.7. Selectivity Determination of Eu@CDs-MIMs and Eu@CDs-NIMs

**Figure 7:**
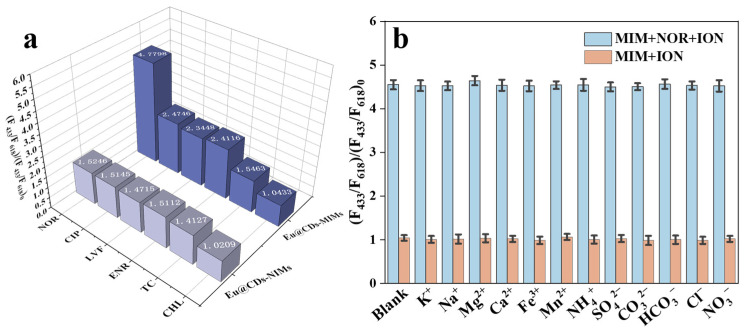
Fluorescence response of Eu@CDs-MIMs and Eu@CDs-NIMs in different kinds of antibiotics (**a**). Fluorescence response of Eu@CDs-MIMs in common ions (**b**).

Selective recognition ability is an important factor for Eu@CDs-MIMs to recognize NOR in complex water environments. In order to investigate the selectivity determination of Eu@CDs-MIMs for NOR, three structural analogues of NOR, namely enrofloxacin (ENR), levofloxacin (LVF) and ciprofloxacin (CIP), along with tetracycline (TC) and chloramphenicol (CHL) as representative antibiotics, were configured to 50 nM of solution for fluorescence detection by Eu@CDs-MIMs and Eu@CDs-NIMs, respectively. As shown in Figure 7a, it can be inferred that Eu@CDs-NIMs lack selectivity in the absence of specific recognition sites from the corresponding fluorescence intensity ratio of Eu@CDs-NIMs between different antibiotics. The increased fluorescence intensity ratio of Eu@CDs-MIMs for CIP, LVF and ENR can be attributed to their similar structure, which enhances their binding ability. It is worth nothing that Eu@CDs-MIMs still has the highest binding ability to NOR. In contrast, the fluorescence intensity ratio of TC and CHL remains unchanged. The results suggest that Eu@CDs-MIMs as a fluorescence test strip can be used for the selective detection of NOR and has the potential to identify a group of structurally similar fluoroquinolone antibiotics.

### 2.8. Interference of Ions on Eu@CDs-MIMs and Eu@CDs-NIMs

The interference from common ions in natural water and wastewater is an inevitable influencing factor in actual detection. SO_4_^2−^, CO_3_^2−^, HCO_3_^−^, Cl^−^, NO_3_^−^, Mn^2+^, Fe^3+^, Ca^2+^, Mg^2+^, Na^+^, and K^+^ were configured to 500 nM of aqueous solution to evaluate the ion interference of Eu@CDs-MIMs in fluorescence detection, as shown in Figure 7b. In comparison to the blank sample, the addition of ions in detection solution has no significant effect on the fluorescence performance of Eu@CDs-MIMs. Subsequently, 50 nM NOR was added to each of the solutions that contained the different ions. The same phenomenon indicates that common ions have no interference in the fluorescence detection of NOR.

### 2.9. Reusability Research on Eu@CDs-MIMs

**Figure 8:**
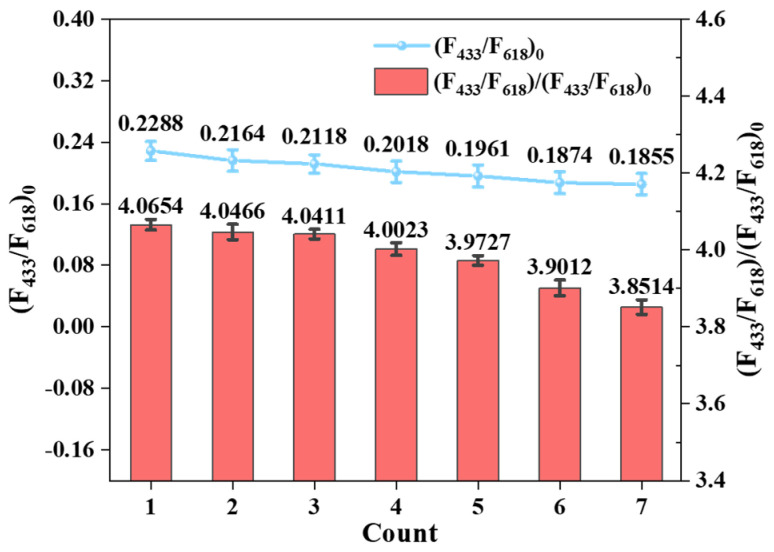
Number of cycles of Eu@CDs-MIMs.

Compared to conventional paper-based test strips, organic membrane materials exhibit excellent chemical and thermal stability along with favorable mechanical properties. The recyclability of Eu@CDs-MIMs was assessed via repeated elution and recombination experiments using (F_433_/F_618_)_0_ and (F_433_/F_618_)/(F_433_/F_618_)_0_ as indicators. The Eu@CDs-MIMs were regenerated using methanol/acetonitrile (*v*:*v* = 4:1) until the fluorescent color no longer changed. The fluorescence measurements were repeated seven times over the same membrane. After undergoing repeated elution and recombination seven times, as illustrated in Figure 8, the initial fluorescence intensity decreases by 19%. However, the relative fluorescence intensity is still retained (94.7%), demonstrating the excellent fluorescence performance of Eu@CDs-MIMs. Therefore, these results indicate that the Eu@CDs-MIMs have good recyclability and the elution process is operationally simple for end users, which can reduce the cost of detection of NOR and the environmental impact.

### 2.10. Fluorescent Test Strip Detection of NOR

**Figure 9:**
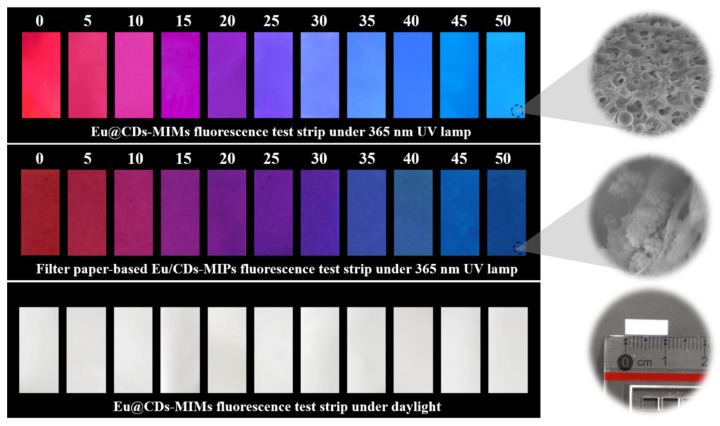
The pictures of a Eu@CDs-MIMs fluorescence test strip and filter paper-based Eu/CDs-MIPs fluorescence test strip with different concentrations of NOR under 365 nm UV lamp and daylight; the inset was SEM images of the surface of the fluorescence test strip.

The visual performance of a fluorescence test strip is the final purpose of this work. The filter paper-based Eu/CDs-MIPs as a comparison to Eu@CDs-MIMs were prepared by the impregnation method, in which the filter paper was immersed in 50 mL of ethanol dispersed with 100 mg of Eu/CDs-MIPs, stirred for 2 h and dried for use. Both test strips were cut to 1 by 0.25 cm and then impregnated with an aqueous NOR solution in the concentration range from 0 to 50 nM for 1 min. Figure 9 shows the pictures of a Eu@CDs-MIMs fluorescence test strip and filter paper-based Eu/CDs-MIPs fluorescence test strip with different concentrations of NOR under 365 nm UV lamp and daylight. Both test strips have the ability to recognize NOR, and as the concentration of NOR increases, the strips show a color change visible to the naked eye. Due to the rough surface of the filter paper and the limitations of the impregnation method, filter paper-based Eu/CDs-MIPs have an uneven color distribution and a poor color-change process. In contrast, PVDF-based Eu@CDs-MIMs exhibit a brighter and more uniform fluorescent color-change process, which is more suitable for visualization. Especially, compared to other visual fluorescent sensors (shown in Table 1), Eu@CDs-MIMs as a fluorescence test strip have significant advantages in terms of detection time and LOD, which can quickly and accurately identify NOR from complex samples.

### 2.11. Smartphone-Assisted Visual Sensing Detection

**Figure 10:**
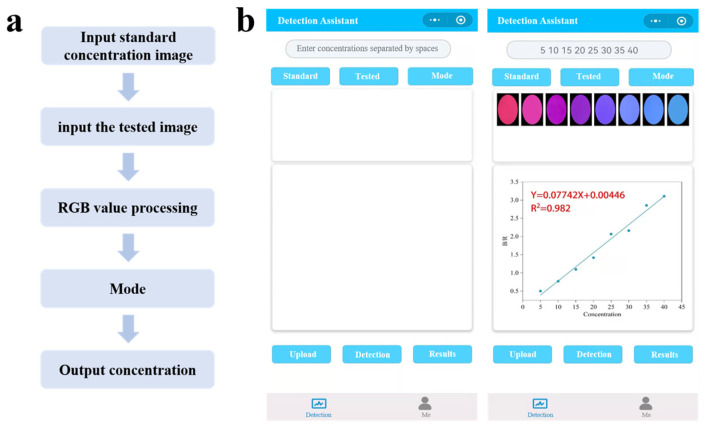
Detection process diagram (**a**) and schematic diagram of detection program (**b**).

Although Eu@CDs-MIMs demonstrate strong concentration fluorescence color dependence, relying solely on naked eye recognition remains inadequate to detect subtle color changes. To improve the performance of quantitative analysis in visual detection, a smartphone was utilized to translate RGB values into numerical-assisted visual detection. To convert RBG values, the test strip was placed in a dark box with a 365 nm UV lamp. A fixed camera parameter mobile phone was used to capture images of test strips and then analyzed using software. The detection steps are performed according to the process shown in Figure 10. First, the images under the standard concentration in the linear range were input to form a standard curve, and the standard curve was established by selecting the corresponding color channel according to the known type of color change in the test strip. Following the input of the image to be tested, the pollutant concentration was determined according to the standard curve. To verify the reliability of the testing software, the images of Eu@CDs-MIMs under a 5–40 nM range were input to create a standard curve, and the standard curve generated by the software has a good linear relationship with an R^2^ of 0.982, and the LOD is determined to be 7.92 nM.

### 2.12. Study on the Mechanism of Fluorescence Enhancement

**Figure 11:**
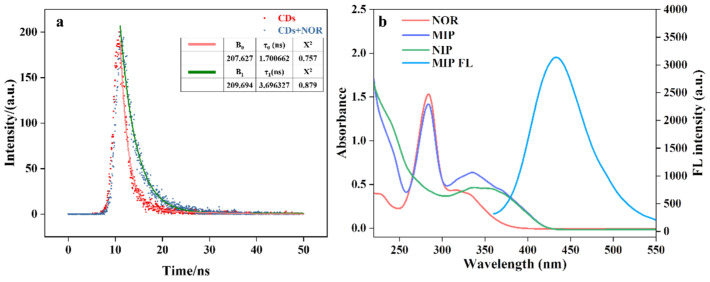
Fluorescence lifetimes of CDs (red square) and CDs with NOR (blue square), the corresponding first-order fitting curves of CDs (pink line) and CDs with NOR (green line) (**a**). Fluorescence emission spectrum of CDs (blue line), UV absorption spectra of NOR (orange), MIPs (purple line) and NIP (green line) (**b**).

To investigate the fluorescence enhancement phenomenon induced by Eu@CDs-MIMs during the detection of NOR, fluorescence lifetime analysis and UV absorption spectroscopy were employed to explore the fluorescence enhancement mechanism between MIMs and NOR. The fluorescence lifetimes of CDs and their complexes with NOR are shown in Figure 11a. After mixing with NOR, the fluorescence lifetime of CDs is significantly enhanced. Meanwhile, the UV absorption spectra of NOR, MIMs, and NIPs (Figure 11b) show that the UV absorption intensity of Eu@CDs-MIMs is significantly increased after conjugation with NOR, but the wavelength of NOR is almost unchanged. It indicates that the fluorescence enhancement of CDs is not caused by the fluorescence resonance energy transfer but by the charge transfer triggered by the conjugation between CDs and NOR [50]. The charge transfer and hydrogen bond have a synergistic effect that facilitates the production of larger chromophore and fluorophore conjugates [49]. Due to the strong hydrogen bonding between CDs and NOR [35], the fluorescence enhancement in this work is based on the synergistic effect of charge transfer and hydrogen bonding between CDs and NOR.

### 2.13. Real Water Sample Analysis

To verify the reliability of Eu@CDs-MIMs for the visual detection of NOR in real water samples, tap water and river water were used to prepare NOR solution to be tested in the concentration range of 5–50 nM for recovery testing. The river water samples were taken from rivers on the campus of Jiangsu University and centrifuged to remove suspended impurities before use. The results of fluorescence detection and HPLC (Figure S5) are shown in Table 2, which demonstrate that Eu@CDs-MIMs exhibit a favorable detection performance across tap water and river water. Additionally, Eu@CDs-MIMs display consistent recovery within the 5–50 nM linear range in all samples. By comparing with HPLC results, Eu@CDs-MIMs demonstrate reliable detection performance, further affirming its ant-interference capability. 

## 3. Experimental Section

### 3.1. Materials

Eu_2_O_3_, ethylene glycol dimethacrylate (EGDMA), polyvinylidene fluoride (PVDF), *N*,*N*-Dimethylacetamide (DMAc), methacryloxy propyl trimethoxyl silane (KH570), methacrylic acid (MAA), and 2-fluoroacrylic acid were all obtained from Aladdin Chemistry Co., Ltd. (Shanghai, China). Citric acid anhydrous, urea, 2,2′-azobis (2-methylpropionitrile) (AIBN), 1,10-phenanthroline monohydrate (phen), polyvinylpyrrolidone (PVP), and norfloxacin (NOR) were purchased from Sinopharm Chemical Reagent Co. (Shanghai, China). All other chemicals used were analytical grade and obtained commercially.

### 3.2. Apparatus

Fourier transform infrared (FT-IR) was obtained on a Nicolet iS50 FTIR apparatus (Thermo Scientific, Waltham, MA, USA). The morphology and sizes were evaluated using scanning electron microscopy (SEM) (JSM-7800F, JEOL, Akishima City, Japan) and transmission electron microscopy (TEM) (JEM-2010HR, JEOL, Akishima City, Japan). The surface morphology of membranes was observed by atomic force microscopy (AFM, Bruker Dimension Icon, Ettlingen, Germany). The fluorescence intensity of the samples was observed by an F-98 fluorospectrophotometer (Shanghai Lengguang Technology Co., Ltd., Shanghai, China). The surface chemical composition was analyzed by X-ray photoelectron spectroscopy (XPS, Shimadzu/Kratos AXIS SUPRA+, Kyoto, Japan). The water contact angle was measured using a goniometer device (OSA60, LAUDA Scientific, Lauda-Königshofen, Germany).

### 3.3. Synthesis of CDs

The CDs were prepared according to the previous work [46]. Firstly, anhydrous citric acid (1.0 g) and urea (1.0 g) were dissolved in 10 mL of deionized water. Then, the mixture was transferred to a 50 mL Teflon-lined autoclave and heated at 160 °C for 4 h. After cooling the reaction to room temperature, the solution was transferred to a semi-permeable membrane bag with a molecular weight of 1000. Finally, the CDs were purified for 48 h, and the product was stored at 4 °C for further use.

### 3.4. Preparation of Eu(MAA)_3_phen

Eu(MAA)_3_phen was synthesized by the coprecipitation method according to previous works [51]. Europium chloride solution (0.25 mol/L) was prepared from high-purity Eu_2_O_3_ by dissolving in concentrated hydrochloric acid (6 mol/L). The product was washed three times with anhydrous ethanol and then dried to obtain solid EuCl_3_·6H_2_O. Subsequently, EuCl_3_·6H_2_O (1.832 g) was dissolved in anhydrous ethanol (50 mL), and MAA (1.31 mL) was added dropwise into the above solution under continuous stirring. After adjusting the pH value of the mixture to 8 using ammonium hydroxide (2 mol/L), phen (0.991 g) was then added. The mixture was stirred at room temperature for 3 h and kept overnight. The precipitate was filtered and washed with anhydrous ethanol for three times and finally dried at 80 °C to obtain Eu(MAA)_3_phen.

### 3.5. Preparation of Eu/CDs-MIPs

Eu/CDs-MIPs were prepared on the surface of SiO_2_@KH570 as a comparison. First, SiO_2_@KH570 (0.1 g) was dispersed with 50 mL of acetonitrile and magnetic stir, CDs (3.0 mg) and Eu(MAA)_3_phen (10 mg) were dissolved in anhydrous ethanol with NOR (0.063 g) and FAA (0.071 g) and then pre-polymerized for 2 h. Then, EGDMA (0.236 mL) and AIBN (0.05 g) were added and placed under N_2_ atmosphere for 15 min. After polymerization, the Eu/CDs-MIPs were centrifuged for 3 min. After this step, polymers were eluted with methanol/acetonitrile (*v*:*v* = 4:1) to remove the NOR.

### 3.6. Preparation of Eu@PVDF

Eu@PVDF was synthesized by the phase transition method. PVDF (4.0 g) and PVP (0.1 g) were added to a round-bottomed flask, which was followed by the addition of Eu(MAA)_3_phen (10 mg). Then, the mixture was mechanically stirred at a constant temperature for 1 day at 25 °C in DMAc (20 mL) and maintained at a constant temperature for 1 d. The casting solution was slowly poured into the scraper plate, and the membrane scraping operation was performed. The phase transition process was finally completed by placing the scraper in water. The prepared Eu@PVDF was soaked in deionized water for later use.

### 3.7. Preparation of Eu@CDs-MIMs

Eu@CDs-MIMs were synthesized by surface imprinting on the surface of Eu@PVDF, in which the imprinted layer was polymerized through carbon–carbon double bonds provided by Eu(MAA)_3_phen. CDs (3.0 mg) were dissolved in anhydrous ethanol (50 mL) with NOR (0.063 g) and FAA (0.065 g) and pre-polymerized for 2 h. After adding Eu@PVDF to the mixed solution, EGDMA (0.236 mL) and AIBN (0.05 g) were added, and the solution was exposed to a N_2_ atmosphere for 15 min to remove oxygen. After stirring at 60 °C for 24 h, Eu@CDs-MIMs were obtained by washing and drying. After this step, Eu@CDs-MIMs were eluted with methanol/acetonitrile (*v*:*v* = 4:1) to remove the NOR. With the exception of the addition of NOR, the Eu@CDs-NIMs were synthesized in the same way as Eu@CDs-MIMs.

### 3.8. Fluorescence Measurements

Eu@CDs-MIMs and Eu@CDs-NIMs were cut to a size of 1.0 cm multiplied by 0.5 cm for the detection of NOR, and NOR solution in the concentration range of 5 to 50 nM was prepared using double-distilled water as a solvent, respectively. The fluorescent test strip was completely immersed in the solution to be tested and reacted completely for 1 min, and the fluorescence spectrum of the test strip was then removed and examined. The excitation wavelength was 344 nm, the slit width was set at 5 nm, and the voltage of the photomultiplier tube was 700 V. The concentrations of solution used for both the evaluation of selectivity and the ionic interference performance were 50 nM, and the test methods were the same. Fluorescent images of test strips for the visualization of performance studies were taken under dark conditions using a small ultraviolet (UV) lamp with an excitation wavelength of 365 nm.

### 3.9. HPLC Analysis of Actual Samples

The chromatographic separation was performed on HPLC (Agilent 1260 Infinity II, Santa Clara, CA, USA) with a Wonda Cract ODS-2 reversed-phase column (250 mm × 4.6 mm, 5 mm). The mobile phase was a mixture of isocratic eluent (pH 2.4) of methanol and purified water (*v*:*v* = 60:40) with a flow rate of 1.0 mL·min^−1^ using a UV detector of 278 nm. Finally, macromolecular impurities were removed from the actual sample using a 0.22 mm filter, and the sediment was removed by centrifugation.

## 4. Conclusions

In summary, a novel fluorescent test strip based on a Eu complexes-doped PVDF membrane for NOR detection utilizing CDs-doped MIPs as fluorescent probes has been successfully synthesized. NOR could enhance the blue emission of CDs through the charge transfer and hydrogen bonding between CDs and NORs, while the red emission of Eu(MAA)_3_phen remained unchanged. With the gradual addition of NOR, the fluorescent test strip showed a color change from red to blue that is visible to the naked eye, resulting in an accurate and rapid detection of NOR with an LOD of 1.35 nM and a rapid response time of 1 min. Excitingly, the combination of the smartphone could achieve numerical-assisted visual detection with a low LOD of 7.92 nM. In addition, to verify the feasibility of the detection method, the HPLC analysis method was used as a reference. Consequently, the designed fluorescent test strip provides a reliable approach for the visual quantitative detection of NOR, which promises to be used for environmental safety detection.

## Figures and Tables

**Table 1:** Comparison between Eu@CDs-MIMs and other visual sensors.

Visual Sensor Name	Target	LOD	Response Time (min)	References
Eu@CDs-MIMs	NOR	1.35 nM	1	This work
MIP-CP	NOR	81 nM	30	[42]
Y^3+^@CdTe QDs	NOR	31.8 nM	5	[43]
B/RCDs@Fe^3+^	NOR	6.84 nM	3	[44]
PAN/ATP/Tb	NOR	16 nM	3	[28]
CdTe@SiO_2_@FMIPs	NOR	3.28 nM	1	[45]
UCNPs-FICS	NOR	6.04 nM	10	[46]
COF_BMTH-HB_	NOR	0.159 μM	1	[47]
DBXPY@Q [8]	NOR	0.72 μM	1	[48]
Tb@COF-Ru	NOR	0.33 nM	1	[49]

**Table 2:** Detection results of NOR recovery in tap water and river water samples.

Samples		Eu@CDs-MIMs			HPLC Method	
	Added (nM)	Found (nM)	Recover (%)	RSD (%, *n* = 3)	Found (nM)	Recover (%)
Tap water	10	9.3	93	3.2	9.8	98
	20	21.2	106	2.9	20.2	101
	30	30.6	102	2.7	29.7	99
	40	41.5	103.8	3.6	39.8	99.5
	50	50.7	101.4	2.0	50.3	100.6
River water	10	10.4	104	3.3	10.2	102
	20	19.6	98	2.9	19.7	98.5
	30	31.5	105	2.6	30.3	101
	40	42.7	106.8	3.1	40.1	100.2
	50	49.3	98.6	2.4	49.8	99.6

## Data Availability

Data are contained within the article and Supplementary Materials.

## Supplementary Materials

The following supporting information can be downloaded at https://www.mdpi.com/article/10.3390/molecules29030661/s1, Figure S1: FTIR spectra of PVDF, Eu@PVDF, and Eu@CDs-MIMs; Figure S2: TEM image of CDs (a) and HR-TEM image of CDs (b); Figure S3: Excitation spectrum and emission spectrum of Eu(MAA)_3_phen (a) and CDs (b); Figure S4: Fluorescence spectra of Eu/CDs-MIPs (a) upon the exposure to different concentration of NOR ranges from 0 to 50 nM. Linear relationship of Eu/CDs-MIPs (b) upon the same concentrations range of NOR; Figure S5: The parameters of HPLC results; (b) linear equation curve for NOR.
