# Supplementary material for: Enhanced Molecularly Imprinted Fluorescent Test Strip for Rapid and Visual Detection of Norfloxacin via a Smartphone
Source: Molecules. 2024 Jan 31;29(3):661. doi: 10.3390/molecules29030661 (PMC10856333; doi:10.3390/molecules29030661)
Supplement: Supplementary file 1 [file molecules-29-00661-s001.zip › molecules-2791963-supplementary.pdf]

Supplementary Materials

Enhanced molecularly imprinted fluorescent test strip for rapid and visual detection of Norfloxacin via a smartphone

Bo Hu [1], Wenshi Zhao [3], Li Chen [2], Yang Liu [3], Zhongfei Ma [1], Yongsheng Yan [2] and Minjia Meng [2,*]

[1] School of Environment and Safety Engineering, Jiangsu University, Zhenjiang 212013, China
2111809001@stmail.ujs.edu.cn (B.H.); mzf@ujs.edu.cn (Z.M.)

[2] School of Chemistry and Chemical Engineering, Jiangsu University, Zhenjiang 212013, China
lchen@ujs.edu.cn (L.C.) yys@mail.ujs.edu.cn (Y.Y.)

[3] Key Laboratory of Functional Materials Physics and Chemistry of the Ministry of Education, Jilin Normal University, Changchun 130103, China
zws6979@163.com (W.Z.); liuyang@jlnu.edu.cn (Y.L.)

* Correspondence: mmj@ujs.edu.cn

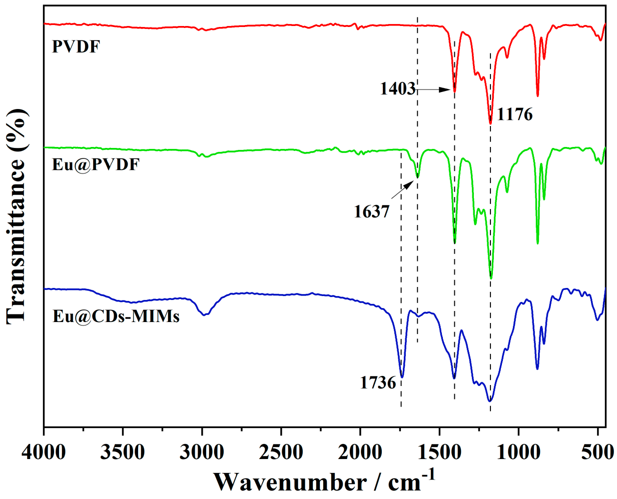

Figure. S1. FT-IR spectra of PVDF, Eu@PVDF, and Eu@CDs-MIMs.

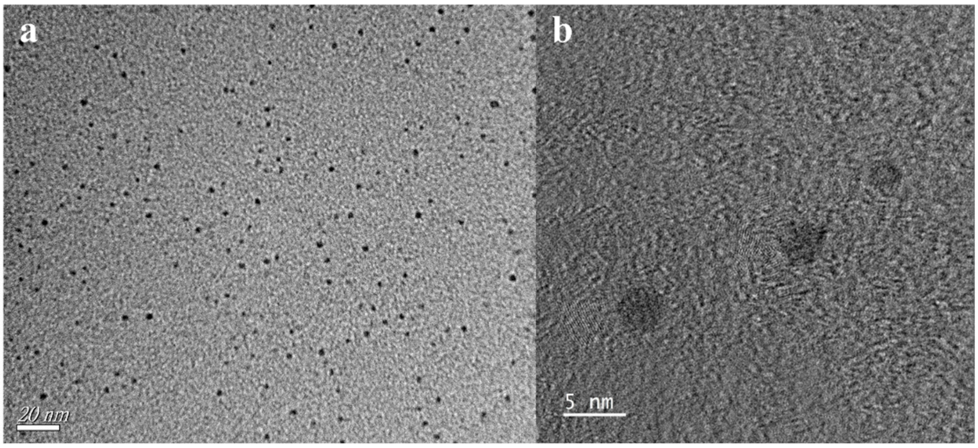

Figure. S2. TEM image of CDs (a) and HR-TEM image of CDs (b).

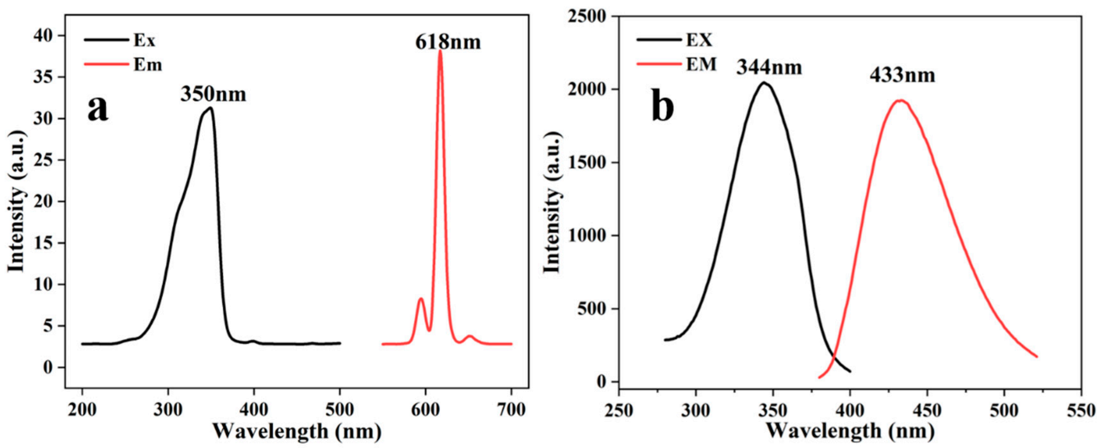

Figure. S3. Excitation spectrum and emission spectrum of Eu(MAA)$_3$phen (a) and CDs (b).

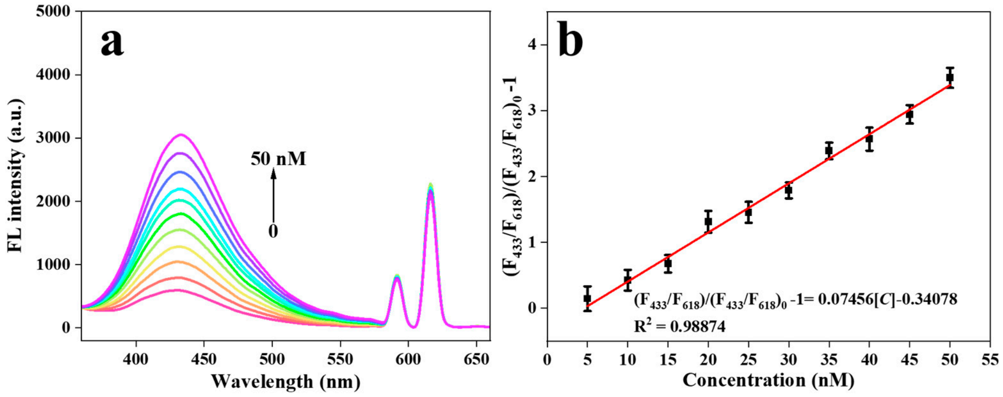

Figure. S4. Fluorescence spectra of Eu/CDs-MIPs (a) upon the exposure to different concentration of NOR ranges from 0 nM to 50 nM. Linear relationship of Eu/CDs-MIPs (b) upon the same concentrations range of NOR.

a

NOR Conc./nM	Retention Time/min	Peak area
5	9.645	26.865
10	9.478	52.316
15	9.706	79.102
20	9.517	101.759
25	9.615	130.163
30	9.461	156.871
35	9.764	185.611
40	9.853	209.403
45	9.623	237.6835
50	9.574	262.85

b

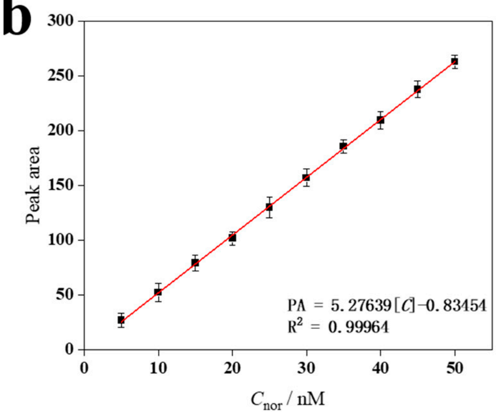

Figure. S5. (a) The paramaters of HPLC results; (b) linear equation curve for NOR.
